# *Notes from the Field:* Rubella Infection in an Unvaccinated Pregnant Woman — Johnson County, Kansas, December 2017

**DOI:** 10.15585/mmwr.mm6740a7

**Published:** 2018-10-12

**Authors:** Tiffany Wallin, Elizabeth Holzschuh, Caitlin Kintner

**Affiliations:** ^1^Disease Containment Division, Johnson County Department of Health and Environment, Olathe, Kansas; ^2^Strategic Planning Division, Johnson County Department of Health and Environment, Olathe, Kansas.

On December 14, 2017, a school nurse notified the Johnson County (Kansas) Department of Health and Environment (JCDHE) that a student’s mother (patient) had received a diagnosis of rubella. The school nurse learned of the patient’s diagnosis when the patient picked up her daughter at school the day of the diagnosis. Follow-up by JCDHE revealed that the U.S.-born patient, aged 27 years, was 19 weeks pregnant and had not been vaccinated against rubella because of personal choice. She had tested negative for rubella by immunoglobulin G (IgG) serology during her first trimester of pregnancy.

On December 6, the patient visited a hospital emergency department complaining of palpitations, a burning, itchy rash, and fever. On December 9, she visited a second emergency department and was told she was having an allergic reaction. After conducting an Internet search, she suspected her symptoms might be caused by rubella and contacted her obstetrician, who referred her to a primary care provider. On December 12, the primary care provider submitted a blood specimen to a commercial laboratory for rubella immunoglobulin M (IgM) testing, which was reported as positive on December 14; the provider informed the patient but did not notify JCDHE.

JCDHE determined the patient had no travel history. When the patient was 15 weeks pregnant (17 days before her rash onset), her unvaccinated U.S.-born brother, aged 22 years, stayed in her home after returning from India, a country with endemic rubella transmission. The brother had a rash on his lower extremities that was diagnosed as poison ivy. Specimens from the patient and brother were collected and submitted to CDC; results were rubella IgG-positive with low avidity, indicating recent infection.

Among approximately 120 contacts of the patient, three were not vaccinated, including the patient’s daughter, aged 11 years, one hospital staff member, and the patient’s female coworker at a call center. All three were advised to avoid contact with pregnant women for 23 days; the patient’s daughter and the hospital staff member were excluded from school and work, respectively, for 21 days.

Rubella infection in pregnancy can result in miscarriage, stillbirth, or congenital rubella syndrome (CRS), which is characterized by low birthweight and birth defects including deafness, cataracts, heart defects, and intellectual disabilities (*1*). The severity and nature of defects depend upon the gestational age of the fetus at the time of infection. The risk for CRS ranges from 10%–90% and is highest when infection occurs during the first trimester (*2*). Endemic transmission of rubella was eliminated in the United States in 2004 as a result of high levels of coverage with measles-mumps-rubella vaccine (MMR) (*1*).

An obstetrician specializing in high-risk pregnancies followed the patient for the remainder of her pregnancy. All follow-up testing was negative, and the patient delivered a full-term, apparently normal, infant in May. Echocardiogram, skeletal survey, head ultrasound, hearing, and eye exams were normal. The infant’s initial rubella IgM was positive, and two sets of nasopharyngeal and urine specimens, obtained 30 days apart, were negative for rubella RNA by reverse transcription–polymerase chain reaction. Until negative results for rubella virus were received, the infant was considered infectious. Based on test results and the absence of congenital defects, indications are that this infant meets the criteria for congenital rubella infection and not CRS (*3*). The infant will continue to be followed by an infectious disease specialist.

This case highlights several important points. Per the Advisory Committee on Immunization Practices recommendations, health care institutions should ensure that all persons working in health care facilities have documentation of adequate vaccination against measles, mumps, and rubella or evidence of immunity (*4*); the hospital staff member who was excluded received the MMR vaccine before returning to work. Health care providers should routinely assess women of childbearing age for evidence of rubella immunity (IgG antibodies) and recommend vaccination when appropriate. Pregnant women testing negative for rubella immunity should be vaccinated immediately after delivery (*4*); this case represents a missed opportunity for rubella vaccination after the birth of the patient’s first child. When a pregnant woman develops a rash illness, providers should ask about international travel for both the patient and her contacts. Finally, more emphasis and education are required for health care providers on the importance of timely reporting of suspected vaccine-preventable diseases.
